# Intraoperative vascular ligation uncovers the role of tumor vasculature in Doege-Potter syndrome: a case report

**DOI:** 10.3389/fonc.2026.1701711

**Published:** 2026-03-04

**Authors:** Lin Du, Kai Wei, Na Li, Yajing Sun, Dongmei Liu, Geng Xu, Tao Yang, Xiuqiang Zhang

**Affiliations:** 1 Department of Thoracic Surgery, Tianjin Fifth Center Hospital, Tianjin, China; 2 Department of Radiology, Tianjin Fifth Center Hospital, Tianjin, China; 3 Department of Emergency, Tianjin Fifth Center Hospital, Tianjin, China; 4 Department of Pathology, Tianjin Fifth Center Hospital, Tianjin, China; 5 Department of Endocrine, Tianjin Fifth Center Hospital, Tianjin, China

**Keywords:** big-IGF2, Doege-Potter syndrome, non-islet cell tumor hypoglycemia, solitary fibrous tumor, vascular

## Abstract

Solitary fibrous tumor (SFT) is a rare mesenchymal neoplasm that may present with non-islet cell tumor hypoglycemia (NICTH), also referred to as Doege-Potter syndrome, primarily due to aberrant secretion of big-IGF2. However, not all big-IGF2-producing SFTs result in hypoglycemia, implying the involvement of additional pathogenic factors. This article reports a case of a 60-year-old male who presented with hypoglycemic syncope secondary to Doege-Potter syndrome. Preoperative imaging identified a large, highly vascularized SFT, approximately 20 cm in diameter, located in the right thoracic cavity. Intraoperative continuous glucose monitoring (CGM) recorded a gradual normalization of blood glucose levels following sequential ligation of three dominant tumor vessels originated from veins- prior to tumor resection - underscoring the essential role of tumor vascular in facilitating big-IGF2 release. Our findings provide the first direct evidence that tumor vasculature is critical for manifesting clinically significant hypoglycemia, thereby complementing established molecular mechanisms such as IGF2 overexpression and impaired pro-IGF2 processing. These insights advance the understanding of NICTH pathogenesis and hold meaningful implications for refining surgical management strategies.

## Introduction

Solitary fibrous tumor (SFT) is a rare, slow-growing mesenchymal neoplasm composed of distinctive spindle-shaped cells, accounting for approximately 2% of all soft tissue sarcomas (1). These tumors most frequently arise in the pleural and abdominal cavity (2). SFTs exhibit striking size heterogeneity at presentation. Reported diameters range from incidental 1 cm nodules to massive lesions exceeding 40 cm, and a direct correlation often exists between tumor dimensions and clinical manifestations. The majority of SFTs grow slowly and remain clinically silent, often being discovered incidentally during imaging studies performed for unrelated indications (1). While the majority of SFTs are typically indolent, roughly 5% of cases present with non-islet cell tumor hypoglycemia (NICTH), known as Doege-Potter syndrome (DPS) - a consequence of pathological big-IGF2 secretion by the tumor (3, 4). The observation that not all big-IGF2-producing SFTs cause symptomatic hypoglycemia suggests the additional modifying factors (5), and the exact pathophysiological mechanisms underlying NICTH development in these tumors have yet to be fully elucidated. Here we present a case of DPS manifesting as hypoglycemic syncope, characterized by a massive, hypervascular tumor with three dominant draining veins. We monitored endogenous glycemic fluctuations intraoperatively by continuous glucose monitoring (CGM) and found that the ligation of tumor’s draining veins induced a gradual rise in glucose levels prior to tumor excision. This observation suggests that tumor size and vascular characteristics represent potential determinants of NICTH development, operating in conjunction with big-IGF2 secretion.

## Case report

A 60-year-old Chinese male with a BMI of 24.2 was emergently transported to our institution following an episode of acute altered mental status. Prehospital emergency medical services documented profound hypoglycemia with rapid neurological improvement following intravenous glucose administration. On detailed history taking, the patient denied any prior history of hypoglycemic episodes, stating that this syncopal event was his first, and reported no characteristic symptoms of diabetes mellitus including polyuria, polydipsia, or polyphagia. He also specifically reported no recent constitutional symptoms such as cough, chest discomfort, and unintentional weight loss, with no change in body weight over the three months preceding admission. The only notable symptom reported within the month prior to presentation was occasional dizziness. The initial diagnostic workup - consisting of a complete blood count, electrocardiogram, cardiac ultrasound, and cranial computed tomography (CT) - showed no significant abnormalities. The liver and kidney function tests ruled out hepatic or renal etiology for the hypoglycemia.

**Figure 1:**
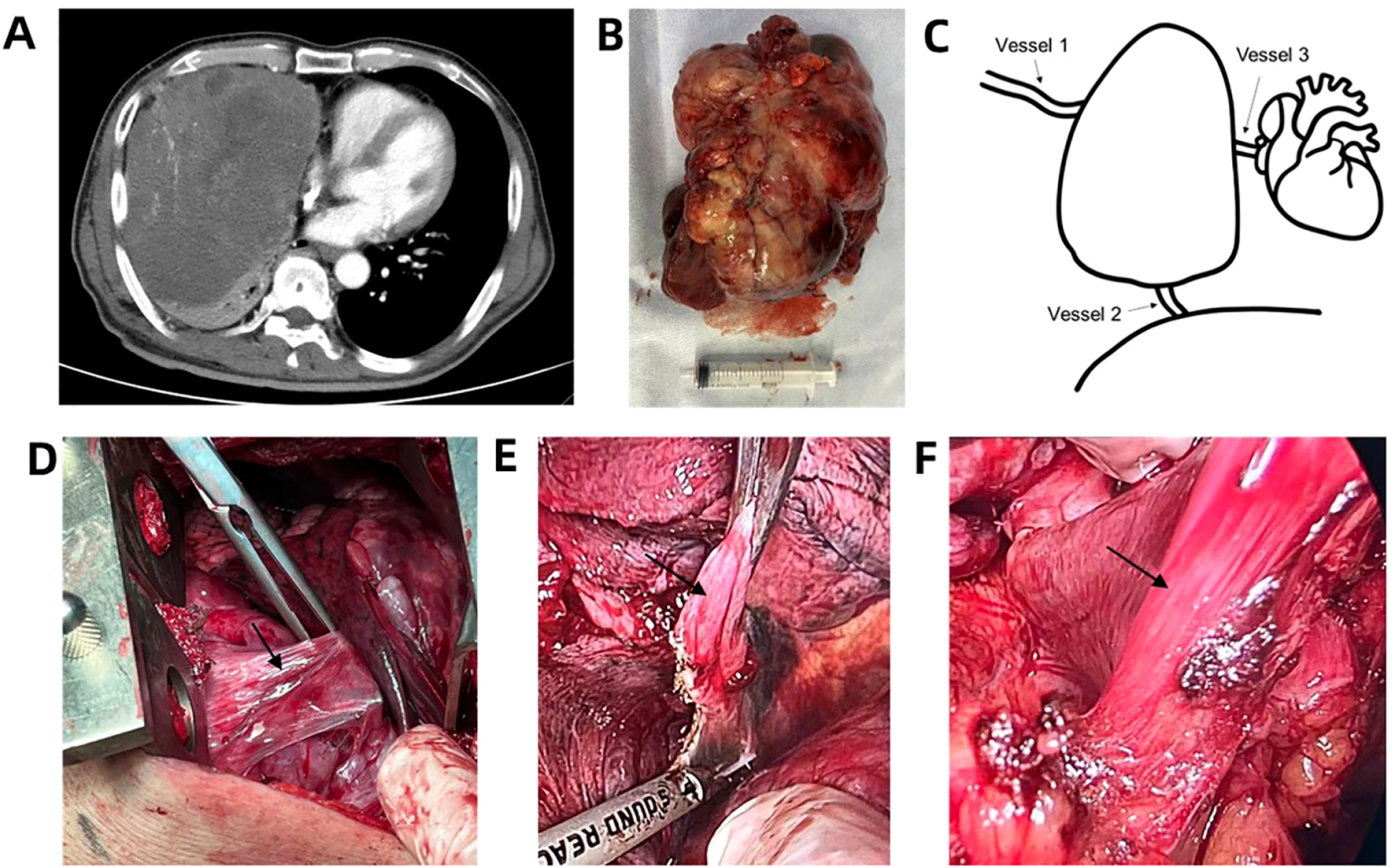
Images of the lesion. **(A)** Contrast-enhanced chest CT revealed a large hypervascular mass in the right thoracic cavity; **(B)** The excised lesion after surgery; **(C)** schematics of tumor and vessels; **(D)** Operative photograph of vessel 1; **(E)** Operative photograph of vessel 2; **(F)** Operative photograph of vessel 3. Arrows indicate the vessels.

Critical laboratory findings included persistently low fasting plasma glucose levels (2.29 mmol/L) with inappropriately suppressed C-peptide (0.032 nmol/L) (**Table 1**) and negative islet cell autoantibodies (GADA, IAA, and ICA antibodies all negative), effectively excluding autoimmune hypoglycemia. Interestingly, tumor marker profiling showed mild elevations in CA125 (72. 80 U/mL; reference 0–24 U/mL), CK19 (4.06 ng/mL; reference 0-3.3 ng/mL), and neuron-specific enolase (NSE 15.6 ng/mL; reference 0-15.2 ng/mL), raising suspicion for a neoplastic process. Chest CT revealed a large hypervascular mass (approximately 16.7 cm) occupying the right thoracic cavity, accompanied with compressive atelectasis and pleural effusion (**Figure 1A**). After multidisciplinary evaluation, surgical resection was indicated.

**Table 1:** Laboratory results pre-/post-resection.

Examination	Preoperation value	Postoperation value	Reference
Glucose (Fasting)(mmol/L)	2.29	5.33	3.9-6.1
C-Peptide (Fasting) (nmol/L)	0.032	0.72	0.37-1.47
Insulin (Fasting) (pmol/L)	–	66.6	17.8-173

**Figure 2:**
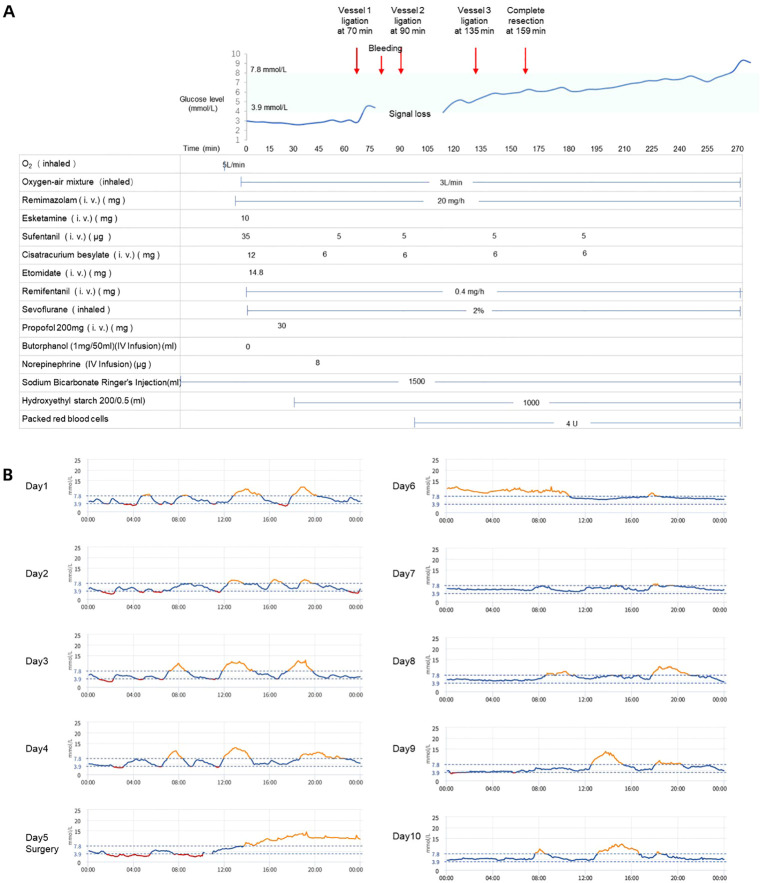
**(A)** Continuous glucose monitoring during surgical procedure and intraoperative medication administration. Red arrows indicate the time points of major vascular ligation events, bleeding and complete tumor resection. **(B)** Blood glucose trends pre- and postoperatively. Surgery was performed on Day 5.

Ten days after admission, the patient underwent a video-assisted thoracic surgery (VATS)-guided right thoracotomy to ensure safe resection of the massive lesion. CGM (Silicon Glycemic Pro, SIBIONICS^®^) was employed throughout the procedure without concurrent exogenous glucose infusion to accurately track endogenous glycemic fluctuations. Prior to surgery, the patient received premedication consisting of intravenous dexamethasone (5 mg), ondansetron (8 mg), desmopressin (20 μg), penehyclidine (0.2 mg), and salbutamol oral spray. Anesthesia induction was initiated (T = 0) with the administration of sedative, analgesic, and neuromuscular blocking agents, accompanied by intravenous infusion of normal saline for fluid support (**Figure 2A**). Active warming, including the use of a forced-air blanket and warmed intravenous fluids, was initiated thirty minutes later to maintain core body temperature within the normal range (36.5–37.0 °C) throughout the procedure. Blood glucose levels remained below 3.9 mmol/L throughout the pre-thoracotomy period (**Figure 2A**).

The surgery commenced approximately one hour after anesthetic induction. Intraoperative findings revealed a 20 cm × 17 cm × 15 cm firm, irregularly surfaced tumor with dense fibrous adhesions to surrounding mediastinal adipose tissue (**Figure 1B**). Dissection was performed using a CSUS6000 ultrasonic scalpel (Reach Surgical) with a CS2305Y blade. The tumor’s draining vessels were derived from three dominant veins with collateral branches (**Figure 1C**): Vessel 1 originated from an intercostal vein (**Figure 1D**), Vessel 2 from a phrenic vein (**Figure 1E**), and Vessel 3 from a vein within the pericardial fat (**Figure 1F**). Vessel 1 was ligated at 70 min. Subsequent to this, significant intraoperative bleeding was encountered; after hemostasis was achieved and a blood transfusion (4 U) was initiated at 90 min (**Figure 2A**). Vessel 2 was ligated concurrently at this time. The final vessel (Vessel 3) was ligated at 135 min, followed by the complete resection of the tumor (**Figure 1B**) at 159 min. The total intraoperative blood loss was approximately 1200 ml.

Before surgery, the patient had a capillary blood glucose level of 2.29 mmol/L, and the CGM yielded a reading of 2.8 mmol/L. These consistent results mutually validated the presence of hypoglycemia in the patient. During the surgical procedure, no additional glucose was administered to the patient. A rapid glycemic surge was observed following the first vessel ligation, suggesting immediate reduction in big-IGF2 delivery (**Figure 2A**). However, data acquisition was temporarily interrupted, labeled as signal loss on **Figure 2A**, most likely due to sensor-skin contact disruption caused by tissue manipulation or direct pressure during the surgical procedure. When monitoring recommenced, the blood glucose reading was comparable to pre-disconnection levels, likely resulting from device calibration. Glucose levels steadily increased again following the third vessel ligation. By the time of complete tumor resection, glucose levels had already been in the normal range (**Figure 2A**). The patient maintained normoglycemia with no episodes of hypoglycemia for several days after surgery (**Figure 2B**), exhibited no postoperative complications, and was discharged accordingly.

The surgical pathology revealed a mesenchymal-origin spindle cell neoplasm exhibiting areas of high cellular density and increased mitotic activity (>4 mitoses per 10 high-power fields). Immunohistochemical analysis demonstrated strong positivity for STAT6, CD34, and Bcl-2, with a Ki-67 proliferation index of 20%. Based on these characteristic histopathological features and immunohistochemical profile, the patient was definitively diagnosed with malignant solitary fibrous tumor (SFT) complicated by Doege-Potter syndrome. Postoperative imaging showed no residual disease, and glucose levels remained normal (**Table 1**).

The patient’s most recent follow-up was at 12 months after surgery. Throughout the entire postoperative period, the patient remained euglycemic with no episodes of hypoglycemia and did not require any adjuvant therapy or glycemic management. Surveillance imaging showed no evidence of tumor recurrence. Notably, the patient gained 15 kg in body weight during the follow-up period.

## Discussion

Intraoperative blood glucose monitoring revealed that tumor vasculature exerts a critical role in the pathogenesis of NICTH. The present case of DPS was characterized by a large, hypervascular SFT featuring three major draining veins. Following intraoperative ligation of these veins, the patient’s blood glucose levels gradually increased and normalized even before tumor excision. Importantly, this restoration of normoglycemia was accomplished without exogenous glucose supplementation during the procedure, indicating that vascular isolation is pivotal for reversing the hormonal abnormalities driving hypoglycemia. This contrasts with a prior DPS case, where intraoperative blood glucose remained stable due to glucose supplementation for hypoglycemia correction (6).

The pathophysiology of NICTH remains incompletely elucidated, though autonomous tumor secretion of incompletely processed big-IGF2 is considered the principal driver (3, 7). This mechanism is supported by consistent clinical observations, including elevated levels of big-IGF2 in serum and tumor tissues, as well as a clinical course in which tumor resection alleviates hypoglycemia alongside declining IGF2 levels, while recurrence restores both conditions (2, 8–10). However, not all big-IGF2-producing SFTs induce hypoglycemia (5). This clinical discrepancy underscores the likely contribution of additional factors in the development of NICTH.

Our intraoperative glycemic trend observed during vessel ligations indicates that tumor vascularization plays an indispensable role in NICTH pathophysiology. Moreover, the tumor has an extensive vascular network with three dominant vessels and numerous collateral branches, forming an efficient conduit for systemic delivery of big-IGF2. These findings are corroborated by histological studies describing characteristic “staghorn” vascular patterns in SFTs, composed of thin-walled, dilated vessels with unique endothelial-tumor cell fusion phenomena (11, 12). Such specialized vasculature not only sustains tumor growth but also enables voluminous endocrine secretion and rapid systemic dissemination of big-IGF2. Furthermore, larger SFTs (>8 cm) have been shown to exhibit higher vessel density compared to smaller tumors (12), providing an anatomical basis for the relationship between tumor size and vascularization.

Furthermore, tumor volume plays a pivotal role in the manifestation of NICTH. Over 96% of NICTH-associated SFTs have a maximum diameter exceeding 10 cm (2, 13), and the tumor in the present case was consistent with this prior finding, with a maximum diameter of more than 20 cm. Tumor volume reduction is the primary approach to ameliorating hypoglycemia. For resectable lesions, for instance, both complete resection and subtotal tumor debulking have been reported to achieve immediate normalization of blood glucose and sustained remission in the absence of recurrence (9, 14–17). In cases of unresectable tumors, transcatheter arterial embolization (TAE) serves as an alternative strategy. TAE induces tumor ischemia and subsequent shrinkage, leading to gradual glycemic improvement over a period of hours to days (18–20).

Thus, the development of NICTH is underpinned not only by an intrinsic molecular basis including IGF2 overexpression (21, 22) and impaired pro-IGF2 processing (5), but also our abovementioned findings further indicate that adequate tumor volume and robust vascularization are indispensable prerequisites for the development of clinical hypoglycemia. Accumulating studies have also underscored the crucial role of vasculature in SFT pathogenesis and immune evasion. For instance, meningeal SFTs have been reported to phenocopy cerebral vascular development and homeostasis and exhibit enriched expression of endothelial markers (23). Furthermore, SFTs exhibit enriched expression of angiogenesis-related receptors and ligands, suggesting that the tumor may facilitate immune evasion or local invasion by mimicking the normal vascular microenvironment (24).

This refined understanding carries important therapeutic implications, particularly for unresectable or metastatic NICTH. Interventions such as targeted embolization of hypervascular regions or subtotal resection of vascular-rich areas may offer effective palliative glycemic control, even when complete excision is not feasible. In addition, our experience together with the previous CGM report (6) supports the value of continuous intraoperative glucose monitoring as a real-time surgical guide and prognostic tool, especially in large or anatomically complex SFTs where the sequence of vascular ligation critically determines metabolic outcomes.

In addition to the intraoperative glycemic changes, we also found a marked increase in glucose levels exceeding 7.8 mmol/L during the early postoperative period, consistent with postoperative stress hyperglycemia, which is a relatively common occurrence (25–29). Surgery, pain, and tissue trauma induce enhanced sympathetic excitation, elevated levels of cortisol, glucagon, catecholamines, and growth hormone, as well as an imbalance in inflammatory cytokine release. These factors collectively promote hepatic gluconeogenesis and glycogenolysis while inducing insulin resistance, ultimately resulting in elevated blood glucose levels (30–33).

This study has two notable limitations. First, IGF2 assay was not performed due to limited laboratory availability for these non-routine tests. This lack of direct biochemical confirmation somewhat limits the definitiveness of the correlation between the tumor and hypoglycemia. Nevertheless, the diagnosis of NICTH was based on three points: (1) documented hypoglycemia, (2) the presence of a large, metastatic solitary fibrous tumor, and (3) resolution of hypoglycemia following tumor-directed therapy in the absence of any other glucose-lowering treatments (34). Second, the CGM-derived glucose values were verified only by a single preoperative fasting capillary measurement, with no further capillary reference values obtained during surgery, particularly the duration of data loss, resulting in an incomplete glycemic record. Nevertheless, the observed CGM trends are unlikely to be artifactual. The sensor was placed remotely from the operative field, and the patient remained hemodynamically and thermally stable throughout. Most importantly, the clear, step-wise glycemic increases were temporally linked to the mechanical ligation of specific vessels and were unaffected by data loss, a pattern inconsistent with random interference or the known physiological delay of CGM, which would shift but not alter the fundamental trend. This supports the validity of the data in reflecting true metabolic changes. The comparable glucose levels observed between the first and second ligations is likely attributable to data interpolation and internal calibration performed by the monitoring device upon signal recovery. Therefore, while the absolute glucose value at this specific time point should be interpreted with caution, the dynamic trend of elevation it presents remains physiologically significant.

## Conclusion

In conclusion, this case underscores the pivotal role of tumor vascularization -alongside established molecular mechanisms - in the pathogenesis of NICTH associated with large solitary fibrous tumors. The real-time glycemic response to selective vessel ligation, observed prior to tumor resection, provides real-time observational evidence that vascular perfusion may play a critical role in the systemic release of big-IGF2 and the consequent hypoglycemia. These findings expand the current understanding of DPS, emphasizing that both tumor size and vascular architecture may act synergistically to mediate clinically significant NICTH. From a clinical perspective, this insight supports the incorporation of preoperative embolization or staged vessel ligation into surgical planning, particularly for large or unresectable SFTs. Further studies are warranted to explore anti-angiogenic and vessel-targeting strategies as potential adjuncts in the control of refractory NICTH.

## Data Availability

The original contributions presented in the study are included in the article/supplementary material. Further inquiries can be directed to the corresponding author.
